# A First Human Case of Ocular Dirofilariosis due to *Dirofilaria repens* in Northeastern France

**DOI:** 10.1155/2011/698647

**Published:** 2011-03-10

**Authors:** Nicolas Argy, Marcela Sabou, Alain Billing, Christian Hermsdorff, Ermanno Candolfi, Ahmed Abou-Bacar

**Affiliations:** ^1^Institut de Parasitologie et de Pathologie Tropicale, Université de Strasbourg et Hôpitaux Universitaires de Strasbourg, 1-3 rue Koeberlé, 67000 Strasbourg, France; ^2^Cabinet de Pathologie, 31 rue du Faubourg National, 67000 Strasbourg, France; ^3^Service d'Ophtalmologie, UF 2406, Hôpitaux Universitaires de Strasbourg, 1 Place de l'Hôpital, 67000 Strasbourg, France

## Abstract

We report the first case of ocular dirofilariasis to be diagnosed in northeast France (Alsace region), in a man who presented with a suborbital mass after a journey to Senegal. Microscopic examination of the surgical specimen identified *Dirofilaria repens.*

## 1. Introduction

Dirofilariasis is a zoonosis occasionally associated with orbital floor infection. The natural definitive hosts are dogs and, more rarely, cats [1–3]. The adult female nematode usually lives in the heart or subcutaneous tissues and sheds microfilariae into the bloodstream [2–4]. The microfilariae are then transmitted by *Culex*, *Aedes*, or *Anopheles *mosquitoes, which are also intermediate hosts [1, 2, 4]. 

Humans are a dead-end host for this nematode, which can cause pulmonary, ocular, or subcutaneous lesions [2, 5–8]. Human orbital dirofilariasis is uncommon. Such patients may present with cysts resembling benign or malignant eye tumors [3, 9]. 

We report the first case of ocular dirofilariasis due to *Dirofilaria repens* to be diagnosed in Alsace, France. The patient presented with an inflammatory periorbital tumor-like lesion.

## 2. Case Report

A 60-year-old man living in Rosheim, Alsace (northeast France) presented with a nodule on the left orbital floor on 8 July 2008. His only recent travel was to Casamance, southern Senegal, in April 2008. Physical examination showed a palpable nodular lesion of the left orbital floor, while magnetic resonance imaging showed an inflammatory nodular lesion. Inflammatory cholangioma, eyelid lymphoedema, or allergies were considered as possible diagnoses. 

**Figure 1:**
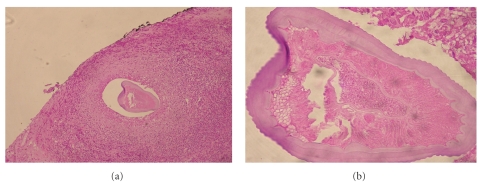
(a) Inflammatory granuloma composed of a polymorphous infiltrate and surrounding a nematode cross-section. (b) Cross-section of *Dirofilaria repens*; note the thick laminated cuticle, external longitudinal ridges, polymyarian muscle fiber, lateral cords, and male gonad.

Antibiotic and steroid therapy had no impact on his symptoms, and the nodule was surgically removed in September 2008. It measured 5 cm along its longest axis. Microscopically, it consisted of polymorphic inflammatory granuloma tissue containing plasmocytes and eosinophilic polymorphonuclear cells, with an epitheliogigantocellular granuloma surrounding a nematode cross-section (Figure 1(a)). The surgical specimen was transferred to our laboratory (Laboratoire de Parasitologie et Mycologie Médicale de Strasbourg) for precise identification of the parasite. The nematode cross-section was composed of a thick laminated cuticle with external longitudinal ridges. A polymyarian muscle fiber was visible, surrounded by a pseudocoelomic cavity. Lateral chords and male gonads were visible in some other sections (Figure 1(b)). Ocular infection by an immature male *Dirofilaria repens* nematode was diagnosed. The patient made a full recovery after surgery, and no further treatment was required.

## 3. Discussion and Conclusion

*Dirofilaria repens* is the most frequent human pathogenic nematode species and is encountered almost exclusively in the old world [1–3, 6]. Most cases have been reported in Italy, followed by France and Greece [1–3, 8]. In France, most cases occur in the southeast, near the Mediterranean coast, where the infection is endemic [1–3, 6, 8, 10]. 

The case of periorbital dirofilariasis reported here involved a man living in Alsace who had stayed in Senegal for three months before symptom onset. The Alsace region is not endemic for dirofilariasis in either dogs or humans [1, 8]. Only three cases of human dirofilariasis have previously been described in Alsace, one of which was shown to have originated in Toulon, southern France [1]. The patient we describe represents the first case of ocular dirofilariasis to be described in the Alsace region. According to Raccurt [10], human dirofilariasis has become more frequent in France over the last twenty years. In 2000, 75 cases of human *Dirofilaria repens* infection were diagnosed in France, three of which were shown to have been acquired in distant regions of the Mediterranean [10]. Dirofilariasis is reported to be emerging in the ex-Soviet Union. Sixteen cases of human dirofilariasis were diagnosed in Hungary between 2001 and 2006, and 15 of the patients had no history of foreign travel. An epidemiological survey conducted in Hungary showed that 14% of dog blood samples were reactive towards *D. repens* microfilariae [7].

Increased international travel in recent decades has favored the emergence of unusual zoonotic parasites such as dirofilaria [5, 6, 8]. Our patient seems to have contracted the infection in Senegal, as he presented with the orbital nodule four months later, in keeping with the reported incubation period of four to eight months [2]. Few human cases of dirofilariasis have been reported in Senegal, but another human case diagnosed in the Mediterranean region of France (Hérault) was also linked to Senegal [10]. Likewise, a patient diagnosed in the United States presented with a periorbital mass due to *D. repens *ten months after returning from a two-year stay in Senegal [11]. Human dirofilariasis is present in Africa, and Senegal is an important reservoir for *D. repens* in dogs and a variety of other animals [6, 11, 12]. The number of human cases of dirofilariasis in Senegal may be underestimated [6]. 

Human *D. repens* infection is difficult to diagnose because of the mild and nonspecific symptoms [2, 3, 6, 8, 9]. Ocular dirofilariasis is more symptomatic [8] but can be mistaken for a bacterial infection, an allergic reaction, or a tumor [3–5]. This can lead to the prescription of ineffective treatments, such as antibacterial agents and steroids [2], as in our patient.

Diagnosis of dirofilariasis is based on a detailed history and microscopic species identification. There are no blood tests for ocular dirofilariasis: eosinophilia is inconsistent, the filarial serology is frequently negative because of low and fleeting antibody production, and tests for microfilaremia are also negative [2, 4, 7, 8].

Species identification of dirofilariae is based on morphological characteristics of the helminth cross-section [2, 5, 8]. *Dirofilaria repens* is characterized by external longitudinal ridges, a thick laminated cuticle, polymyarian muscle fibers, and the diameter and laterals cords [7, 8]. Cross-sections are usually surrounded by polymorphic inflammatory granuloma tissue [3, 8], as in our patient. Alternatively, the nematode may be surrounded by fibrosis, which can alter the parasite morphology and hinder diagnosis [8]. 

Treatment of ocular dirofilariasis consists of excision biopsy, antihelminthic drugs being ineffective [2, 3, 5, 9]. Orbital involvement is uncommon in humans. Diagnosis is hindered by poor awareness of this disease outside enzootic areas and by confusion with other tumoral pathologies. Given the increase in the frequency of this zoonosis in recent years, practitioners should bear in mind the possibility of ocular dirofilariasis when a patient presents with an orbital tumor-like growth or inflammatory lesion after a trip to an endemic region.
